# Diversity in maintaining health of populations

**DOI:** 10.4102/hsag.v27i0.2137

**Published:** 2022-12-13

**Authors:** Bonisile S. Nsibandze

**Affiliations:** 1 Department of General Nursing, Faculty of Health Sciences, University of Eswatini, Mbabane, Eswatini

## Introduction

Multiple interventions can be employed to ensure the health of populations, ranging from those that address the environment to those that address the human being. The human being is seen holistically in interaction with the environment (University of Johannesburg 2010), and therefore to ensure health of the human being, it remains important to study and change the environment with which the human being interacts. Interpersonal interactions and the environment – which is classified as either internal or external (University of Johannesburg 2010) – have a huge effect on human health. The environment may represent the most critical contributor to health disparities.

Health disparities are defined as preventable differences in the burden of disease, injury and opportunities to achieve optimal health (Centers for Disease Control and Prevention 2022). Health disparities exist in a wide spectrum within populations and are heightened by race, gender and socio-economic status (Price, Khubchandani & Webb 2018). In this collection, a wide variety of manuscripts were submitted that seek to identify and address the differences in health status among populations. A majority of manuscripts within this collection were received from researchers in the Limpopo and KwaZulu-Natal provinces.

## Health of populations across the age spectrum

Determining the health of children remains critical to ensure proper growth and development. Malnutrition occurring in early childhood is associated with disturbances in physical growth, including brain development (Onis & Branca 2016), that impact the child’s future (Mwene-Batu et al. 2020). One study looked into the feeding practices and micronutrient status of children aged 0–36 months. This age group usually suffers from malnutrition secondary to multiple factors, not limited to insufficient dietary intake, unavailability of food and uneven distribution of food within families (De & Chattopadhyay 2019). Hence, early screening for malnutrition is key in supporting the growth and development of children, particularly in low socio-economic settings.

## Maintenance of health among employees

The employees’ space has been associated with increased maladies because of the nature of various jobs. A wide range of occupation-associated maladies has been reported, including musculoskeletal issues, loss of functioning senses, obesity secondary to sedentary work position and anxiety and depression because of high-stress work environments (Harshana 2018). Hence, job health maintenance remains a critical point as it is associated with improved health, increased productivity and reduction in sickness-related absence (Song & Baicker 2019). Healthy, safe and regulated work environments are key in ensuring the safety of employees, as well as improving staff morale (Harshana 2018). There are, however, barriers to the implementation of health and safety interventions in the workplace. These include high-pressure environments, financial constraints and reluctance to invest in employee health and well-being (Quirk et al. 2018).

Multiple manuscripts were relevant to the subtopic of employee health maintenance. The first article focused on the experiences of school nurses providing care, and the key findings indicated that unavailability of resources – vehicles, medication, scheduling and limited supportive supervision – hugely impacted their work, which left nurses frustrated. Lack of proper work equipment has been shown to reduce staff morale and further contribute to occupational stress (Harshana 2018). Administrative issues highlighted by this article indicate that populations are disadvantaged and are not receiving preventive and curative services, which indirectly contributes to and perpetuates health disparities within a community of school-going children.

The second article focused on the anthropometric measures for nurses across all qualification tiers. A majority of nurses were found to have a higher body mass index (BMI), along with chronic conditions. Longer working shifts and lifestyle issues such as the use of tobacco and a sedentary lifestyle impacted the overall health status of the nursing workforce who participated in this study.

The last manuscript focused on working conditions in Limpopo and was aimed at preventing infirmities in the workplace. The negative effects of unsafe work environments were identified in this article. Environments that promote abnormal ergonomics may lead to musculoskeletal disorders such as chronic backache. Recommendations were for employers to provide personal protective equipment and train employees in its proper use. Other conditions employees have been shown to experience include sunburns, some degree of hearing loss and harassment by the public.

## Social ills that lead to ill health among populations

According to the University of Johannesburg (2010), the external environment consists of social, physical and spiritual dimensions. The social dimension recognises the human resources in the external environment of a person (University of Johannesburg 2010). This means that the availability of resources in the external environment or lack thereof has the potential to affect an individual’s disease burden. One article addressed the social elements of gender-based violence. Emotional abuse was the central theme of this article. Gender-based violence is defined as a wide spectrum of gender-related violent acts that have roots in power structures that seek to enforce appropriate masculinity or femininity (Mannel & Hawkes 2017) and are associated with a wide range of effects on the victim. As such, this article addressed one form of gender-based violence, namely violence against women in domestic settings. Mental health issues weigh heavily as the outcome of gender-based violence, as illustrated by the article to include negative effects on self-esteem and loss of intimacy. Because of the cycle of abuse against women, multiple reasons have been documented, such as feelings of love for the abuser, lack of financial resources and/or support and considerations for children.

In another study, other social ills have been identified as a major contributor to nonadherence to treatment among clients on lifelong therapy. The study depicted the cycle of health–illness that ensues as a result of multiple patient factors. The effects of poverty also contribute to missed clinic and hospital appointments that in turn lead to poor adherence. Poor adherence to antiretroviral therapy (ART) is strongly associated with poor treatment outcomes among patients living with HIV (Moosa et al. 2019). Financial implications have also been identified as a factor in adherence to ART, in the sense that patients consciously chose to remain with a detectable viral load and suboptimal treatment outcomes for the purpose of receiving social grants from the government.

## Conclusion

The manuscripts reviewed indicate that health disparities occur across populations, and to reduce them, multiple interventions within public health have to be put in place. Financial resources, however, seem to be an anchor in a majority of the health issues. The social environment with which individuals interact with is also closely linked to health disparities. Addressing issues of mental health, particularly for persons who have experienced abuse, is also critical.
